# Generational differences in healthcare: the role of technology in the path forward

**DOI:** 10.3389/fpubh.2025.1546317

**Published:** 2025-02-26

**Authors:** Costanza Cecconi, Rob Adams, Antonella Cardone, Joséfine Declaye, Mitchell Silva, Tineke Vanlerberghe, Nick Guldemond, Ignaas Devisch, Joris van Vugt

**Affiliations:** ^1^Viatris, Rome, Italy; ^2^Six Fingers, Eindhoven, Netherlands; ^3^Cancer Patients Europe, Brussels, Belgium; ^4^European Specialist Nurses Organisation, Brussels, Belgium; ^5^Esperity & Patients Centric, Brussels, Belgium; ^6^Viatris, Hoeilaart, Belgium; ^7^Leiden University Medical Center (LUMC), Leiden, Netherlands; ^8^Ghent University, Ghent, Belgium; ^9^Viatris, Amstelveen, Netherlands

**Keywords:** generational differences, digital health literacy, hybrid healthcare models, artificial intelligence (AI) in healthcare, preventative health

## Abstract

Healthcare systems worldwide are under increasing pressure due to aging populations, rising prevalence of chronic diseases, and heightened patient expectations. Generational differences significantly impact perceptions of health, healthcare decision-making, use of digital technologies, and attitudes toward preventative health. This perspective article explores these differences through the lens of Generational Cohort Theory, focusing on six generations: the Silent Generation, Baby Boomers, Generation X, Millennials, Generation Z, and Generation Alpha. We highlight how each cohort's unique experiences shape their healthcare values, preferences, and engagement with digital health technologies. Younger, tech-savvy generations demand personalized, data-driven healthcare solutions, while older generations often face barriers to adopting digital tools due to limited digital literacy. The article emphasizes the importance of tailoring healthcare delivery, including hybrid care models, to accommodate diverse generational preferences. It further addresses the role of artificial intelligence, wearable technologies, and mobile health apps in preventative care and the need for targeted education to bridge the digital divide and combat misinformation. We propose strategies to integrate digital health solutions and generationally sensitive communication approaches, ensuring equitable access to healthcare services and fostering patient empowerment. Ultimately, this work underscores the need for a multi-faceted, inclusive approach to healthcare delivery to meet the demands of an evolving patient demographic and drive progress in public health systems.

## 1 Introduction

Healthcare systems are under increasing pressure due to rising prevalence of chronic conditions, increasingly aging populations, and fiscal constraints (1). Innovative technologies promoting preventative health interventions and personalized data-driven care represent important opportunities for progress. However, there is no one-size-fits-all approach to integrating these innovations into routine healthcare for diverse patient populations.

**Figure 1:**
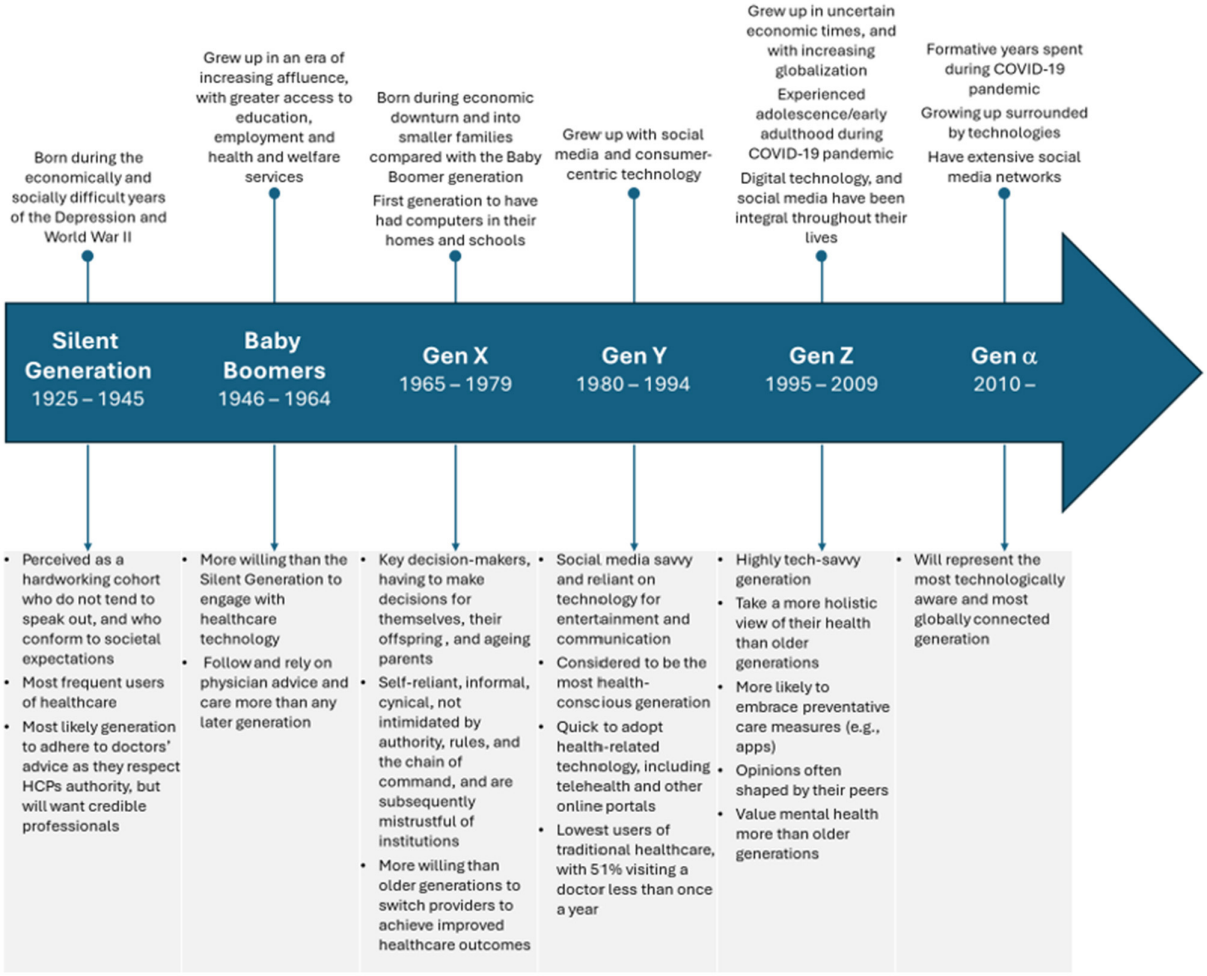
Generational influences and characteristics (2–4, 7–9, 11, 12, 14, 34).

The Generational Cohort Theory describes how individuals from a particular generation tend to have similar behaviors, values and perceptions that are shaped by similar life experiences and witnessing common historical events (Figure 1) (2, 3). The time span (15–25 years) used to define generations has evolved over time, and may be influenced by birth rates, social changes and trends; additionally, the birth year range for different generations may vary subtly according to the source (2–5). Six contemporary generations exist: the Silent Generation (or “Traditionalists”; born 1925–1945); Boomers (1946–1964); Gen X (1965–1979); Gen Y (or ‘Millennials'; 1980–1994); Gen Z (1995–2009); and Generation Alpha (2010–2024) (4). Older generation (≥65 years) represent a rapidly increasing proportion of the population and is expected to rise from 10% in 2022 (i.e., Silent Generation and some Boomers) to 16% in 2050 (i.e., Boomers, and Gen X) (6).

In this perspective article, we explore generational differences in perceptions and expectations of healthcare, and propose suggestions for how to meet diverse generational needs (summarized in Table 1).

**Table 1:** Overview of health-related information source, expectations, challenges, and opportunities by generation.

**Generation (4, 34)**	**Healthcare information sources (3, 9, 11, 12, 34)**	**Expectations/values (3, 4, 8, 9, 12, 18, 21, 34)**	**Challenges (3, 4, 9, 12, 14)**	**Opportunities (3, 8, 12)**
Silent generation (“Traditionalists”), 1925–1945	•HCPs	•Typically prefer traditional healthcare •Less comfortable with telehealth and other online components of medical care	•Misinformation •Online security •Ability to conduct tasks online	•Make more comfortable with the role of digital health technology while maintaining strong interpersonal connection
Baby boomers, 1946–1964	•HCPs and pharmacists •Internet	•Practices with good reputations because they want a doctor they can trust; often choose a practice with a name that they recognize and trust	•Misinformation •Online security •Ability to conduct tasks online	•Leverage openness to technology to provide improved care with progressing age
Gen X, 1965–1979	•Rely on multiple sources of information, including family, colleagues, HCPs, pharmaceutical companies, medical journals, TV, Internet, books. Doctors are preferred source	•Convenience and accessibility due to demands of work/family life (e.g., after-work hours, weekend appointments) •Evidence-based recommendations	•Misinformation •Privacy/security	•Offer convenient appointment times •Online booking portals
Gen Y (“Millennials”), 1980–1994	•Heavily reliant on digital technology and social media •Often rely on their peers' input and patient testimonials when selecting a HCP	•Virtual appointments •Want quick answers and instant gratification as juggling working lives with care-giving responsibilities	•Misinformation •Low use of traditional healthcare	•Convenient methods to make medical decisions online, and foster deep, genuine connections with physicians
Gen Z, 1995–2009	•Social media •Videos	•Value speed, moving quickly from one task to another •Visual engagement (i.e., prefer watching videos to reading articles •Mental health •Convenient digital options (e.g., ability for online message/chat with HCP)	•Misinformation •Perceived as the most anxious generation as a result of an over-protected upbringing	•Digital options and preventative care with an emphasis on mental health and overall wellness •Offer personalized, data-driven health •Offer integrated and holistic approach care (e.g., where mental health professionals are embedded in primary care setting) •HCP presence on social media to mitigate misinformation
Gen Alpha, 2010–2024	•Social media	•Emerging technologies like AI for health and wellness •Personalized services •Mental health	•Misinformation •Over-reliance on technology may result in reduced attention spans and social difficulties •Anxiety is thought to be more prevalent among this cohort that previous cohorts •Are likely to experience more health problems than other generations, predominately because of increasingly sedentary lives	•Collaborations with social media influencers •Preventative care measures •Mental health apps

## 2 Generational differences regarding health

### 2.1 Perceptions of health

The Silent Generation view health traditionally, focusing on physical well-being and the absence of illness, associating healthcare with medical treatment rather than prevention. Baby Boomers also prioritize physical health but are more open to holistic perspectives, though both groups experience greater mental health stigma than younger generations. Generation X take a more holistic view, shaped by technological advances and awareness of mental health and wellness practices (e.g., yoga, alternative therapies). Millennials, the most health-conscious generation, adopt a holistic view of health, emphasizing sleep, nutrition, fitness, mindfulness, and appearance (3, 7). Gen Z and Gen Alpha also have a holistic view of health, and are more willing to talk about mental health as they perceive less stigma associated with having mental health issues–possibly a result of growing up during the COVID-19 pandemic (8, 9).

### 2.2 Healthcare decision-making

In recent decades, patient autonomy has gained emphasis, empowering individuals to take an active role in health decisions. Medical paternalism, where healthcare professionals made decisions without explicit patient consent, has been replaced by patient-centric approaches. Informed decision-making allows patients to decide based on a medical intervention's risks and benefits, while shared decision-making includes input from healthcare professionals, aligning with patients' needs and goals (10).

Access to the internet, social media, preventative health apps, and direct-to-consumer tests has enhanced patient autonomy. Increased patient organizations also empower and advocate for individuals. As a result, younger generations increasingly rely on alternative resources instead of healthcare professionals for medical advice.

The Silent Generation are most familiar with the traditional healthcare model in which doctors are key decision-makers for patients, and are the most likely generation to have long-term relationships with their HCP and to adhere to doctors' advice as they respect HCPs authority (7, 11, 12). Boomers seek information on medications, practical care and nutrition or exercise from HCPs, and are also likely to follow HCP advice (7). They may use the internet for information about symptoms, prognosis and treatment options (11). Generation X tend to be skeptical of large institutions, including healthcare, because of formative experiences (e.g., AIDS crisis) fueled a mistrust in healthcare systems (3). They seek health information from varied sources, including family, colleagues, HCPs, pharmaceutical companies, medical journals, TV, and the internet (3). HCPs are Gen X's preferred source of information, and they have more trust in physicians than Millennials (3). Despite being considered the most health-conscious generation, (3). Millennials have low usage of traditional healthcare systems, with 51% visiting a primary physician less than once per year (12). For advice regarding HCPs, they look to patient testimonials and peers' input (12). Gen Z are more likely to share their health information with non-primary care providers, such as health insurer, retail clinic, or a third-party app (8). They are more likely to trust healthcare information on the internet than from HCPs, but they may also consult their parents when making health-related decisions (13). Social media is also an important source of healthcare information for Gen Z (42% vs. 20% for non-Gen Z generations) (8). Gen Alpha are a highly technically-literate generation growing up with greater access to technology (e.g., smartphones, tablets, virtual reality headsets), information and external influences than previous generations (4). As many Gen Alpha spent their formative years during the COVID-19 pandemic (14), they often spend a lot of time online and have extensive social media networks, which has provided them with more global influences than other generational cohorts (9).

### 2.3 Use of digital technologies

Individuals born after 1980 (i.e., Millennials and Gen Alpha) are considered “digital natives”, having grown in a world defined by the digital technology and the internet, and with digital experiences forming an integral part of everyday life. Those born before widespread digital technology use are called “digital immigrants” as they have had to adapt to using digital technology later in life (3, 15).

The Silent Generation are considered the least comfortable with technology as it was introduced when they were beyond middle ages (4). Their willingness to embrace new technologies may also be influenced by limited finances, or by reserved spending that stems from growing up during a time of austerity (4). A multi-generational study reported that the Silent Generation found mobile technology to be more complex than the youngest generation (15). Research indicates that only 34% of 65–74-year-olds have at least basic digital skills, compared with 69% of 25–34-year-olds (16). This cohort may be more likely to experience information theft or privacy breaches due to uncertainty about appropriate measures to mitigate against such issues (3). A 2022 study by Age UK found that 46% of over-65s (i.e., Silent Generation and some Boomers) could not complete eight key tasks to use the internet safely and successfully (17).

Boomers also reached adulthood without digital technology, but they are more willing to engage with it than the Silent Generation, although more reluctant than younger generations (4, 12). Generation X grew up during the transition between analog and digital, witnessing the introduction of home computers, mobile phones, and the internet. They are comfortable with using digital technology and embrace its convenience. Millennials grew up with the internet and social media and are heavily reliant on digital technology and social media for health-related information; research indicates that Millennials are more likely to own a smartphone than individuals from Gen X or the Silent Generation (15, 18). As digital technology and social media have been integral throughout their lives, Gen Z and Gen Alpha are highly tech-savvy, and also heavily rely on digital tools and social media (4, 8).

### 2.4 Attitudes regarding preventative health

Preventative health uses interventions like exercise, vaccinations, and screenings to prevent or detect disease early, rather than treating it later. Strategies now include wearable devices and health apps that monitor parameters like blood pressure, glucose, and activity. Genomic screening identifies disease predisposition, while AI and machine learning increasingly predict risks and offer personalized care plans (19). The attitudes to, and use of, preventative health varies between generations.

The Silent Generation, less exposed to fitness and wellness trends, are less likely to embrace preventative health, though some adopt it later in life. Boomers are more proactive, engaging in measures like screenings, healthy diets, and exercise as they age. Gen X, shaped by the 1980s fitness culture, value self-care, often incorporating fitness routines, alternative therapies, and stress management. Millennials and Gen Z prioritize preventative care, investing in fitness, nutrition, mental health, mindfulness, and self-care advocacy (8, 13).

### 2.5 Healthcare values and preferences

Each generation has different values and preferences regarding healthcare. The Silent Generation typically prefer traditional in-person medical care and scheduling appointments by telephone or in person (12, 20). Boomers value health practices with good reputations, often choosing practices with names they recognize and trust (12). Gen X value transparent communication, including evidence-based recommendations, from their HCPs (3, 7, 12); convenience is highly valued as many Gen Xers are juggling work with child-rearing and care of older parents (3, 7). Privacy and data security are important to this generation (3). Millennials are also juggling their working lives with care-giving responsibilities, are heavily reliant on digital technology and social media, and would prefer to have a virtual appointment than see a doctor in person (3, 7, 18, 21). They value on-demand (e.g., access to personal health records, online appointment scheduling) and personalized data-driven services (7). Cost is an important factor for Gen Z. Their strong reliance on technology in their day-to-day lives means they expect personalized, data-driven healthcare that offers greater flexibility and convenience than traditional healthcare services (13, 22).

## 3 Challenges to tackle

### 3.1 Addressing generational health needs

Healthcare usage and medical needs vary between different generations, with the Silent Generation using healthcare more than younger generations (11, 12). Essential healthcare services (e.g., type 1 diabetes care) remain consistent across generations, but diverse expectations require tailored approaches. These include offering options for consultations (in-person or virtual), communication methods (phone, email, online portals, social media, chatbots), and flexible clinic hours (e.g., same-day or after-hours care). Younger generations also expect integrated services combining chronic, preventative, and holistic care.

### 3.2 Digital health literacy

Health literacy relates to the ability of an individual to make an informed decision about health-related issues based on the available information (1). Increasing health literacy empowers individuals to take a more active role in improving their health; higher levels of health literacy are associated with better health outcomes and lower levels of health inequalities (23). Digital health literacy is a more complex extension of health literacy, and is broadly defined as the ability to find, appraise, and use health information via technology (1, 24).

While technological advances and the use of the internet and social media offers the potential to broaden the reach health information, it has resulted in a greater health divide. Individuals with higher levels of digital literacy are better able to coordinate health care needs via patient portals, have better involvement in medical decision-making, and improved health outcomes (10, 24). In rural and underserved settings, lack of reliable internet access represents a significant barrier to use of digital health information and resources, and can result in the exclusion of certain sections of society (25, 26). The so-called “digital divide” stems from the inability or unwillingness of some individuals to use or access health information that is available via digital health tools; however, it also reflects the ability of individuals to distinguish high-quality information from information that is incomplete or inaccurate (24, 27).

Misinformation represents a significant obstacle to patient empowerment as misinformed individuals can have persistent beliefs and make poor health-related choices (e.g., refusing vaccination because of misinformation about side effects) (27). Poor digital literacy, often due to limited access to digital resources, is more common among older individuals. However, difficulty distinguishing accurate from inaccurate health information affects all generations, including tech-savvy ones reliant on technology. Misinformation and weak patient-HCP relationships can lead to entrenched, poorly informed views and poor decisions. Enhancing digital health literacy is crucial for empowering patients and combating misinformation (27).

## 4 Opportunities

### 4.1 Digital enablement in healthcare

Digital technologies have a pivotal role to play in improving healthcare access and outcomes for patients, and in improving efficiencies for healthcare systems struggling to manage aging generations with an increasing prevalence of chronic diseases, and younger generations with high expectations for personalized, data-driven care (8, 24). Digital enablement in healthcare relates to the use of digital technologies (e.g., patient portals, virtual consultations, remote monitoring, healthcare apps and wearables, therapeutic virtual reality, AI and machine learning), to enhance and streamline service delivery and improve patient outcomes.

Digital enablement facilitates the improvement of service provision across healthcare, including hybrid healthcare models that allow patients a range of interaction options, and AI-powered tools that help diagnosis and management, as well as tools enhancing preventative and mental health care. The integration of digital technologies also enables multi-channel communications, promotion of health literacy, expanded service availability, the facility for online payments, and easy access to data–features that are key to engaging patients from various generations (3, 13).

### 4.2 Hybrid healthcare models

Hybrid healthcare models address generational preferences by offering options like traditional communication methods for the Silent Generation (e.g., phone scheduling or receiving test results). Combining physical consultations with online tools can improve patient engagement across generations. Research shows Gen X and Millennials often experience poorer physical and mental health and less healthy lifestyles compared to earlier generations (28, 29). Both Gen X and Millennials are juggling work with care-giving responsibilities and value more convenient means of booking appointments (e.g., online booking system) and the ability to submit queries via an online portal (3, 12, 18, 21). Meanwhile, Millennials see themselves as being too busy for face-to-face HCP consultations and value convenience, preferring chats to HCPs on social media and virtual appointments (13). Similarly, Gen Z want digital options such as virtual appointments, the ability to chat online to HCPs, and easy access to their data (3, 13). Hybrid models may also help to foster patient-physician relationships, which are particularly important for young Gen Z and Gen Alpha patients entering the healthcare system alone for the first time (30).

### 4.3 AI-powered healthcare tools

AI is having a growing impact in healthcare. Using techniques such as machine learning and deep learning on large datasets, AI can help to diagnose diseases, develop personalized treatment plans, and assist clinical decision-making (19). AI can also provide virtual health assistance via chatbots and other tools, helping patients to identify underlying health issues based on symptomology, providing medical advice, reminders regarding medications, scheduling doctor appointments, and monitoring vital signs (19). AI-powered mental health tools are another emerging application, assisting in early detection, diagnosis, treatment and support of conditions (e.g., depression).

Another key AI application is patient education, whereby AI-powered chatbots support patients across healthcare scenarios, including dietary advice, smoking cessation, and cognitive behavioral therapy. Importantly from a generational perspective, AI can provide information that is tailored to patients' literacy levels and health status. AI tools can help to reduce HCP workload, increase healthcare access, mitigate human error, and improve patient outcomes (19). However, there needs to be greater awareness among both HCPs and their patients about the role of AI in healthcare applications (e.g., medical diagnoses). Explainable artificial intelligence (XAI) has an important role in providing transparency about the decision-making processes used by AI systems, and helping to increase credibility about AI among HCPs and patients (31).

### 4.4 Increasing health literacy and bridging the digital divide

Low user acceptance is a key barrier to the Silent Generation adopting digital health technology. According to the European Society of Cardiology e-Cardiology Working Group, many digital developments for patients with coronary heart disease have not been developed based on the needs and expectations of patients (32). It is therefore essential to keep the target patient in mind when designing digital health resources (3).

Targeted education can help to improve digital skills among older generations. This should aim to increase awareness about the potential benefits of digital technologies (e.g., providing easy access to test results and to urgent advice, enabling repeat prescription requests), competency in performing basic tasks, and awareness of appropriate measures to mitigate against theft or privacy breaches (3, 7, 12, 22, 33).

Generational gaps in health literacy can be mitigated through targeted education. A holistic approach is needed to providing patient information that considers generational needs of patients, including preferred sources, education and health literacy levels (10). Communications can be tailored to meet generational needs, using clear and accessible language to reduce the risk of misunderstanding, considering age-appropriate health messaging (e.g., cervical cancer screening for Gen X and Baby Boomers) and target cohorts' communication preferences. The Silent Generation and Boomers prefer in-person information or printed materials, while email, website and social media platforms (e.g., Facebook) are preferred by Gen X. Millennials and Gen Z prefer social media and mobile apps, and are more likely to watch a video than read an article about a health issue (34). Building community partnerships may help to extend engagement with health literacy strategies beyond the clinical setting (10).

Tackling misinformation will also be essential to bridging the digital divide. Data from 2019 showed that Google received ~1 billion health questions every day (1), while health-related videos were viewed more than 110 billion times globally on YouTube in 2021 (35). Greater collaboration is needed between tech providers (e.g., social media companies) and healthcare organizations to tackle misinformation (36). HCPs role is vital to countering misinformation but will only be successful via collaborative and trusting HCP-patient relationships (27). Providers can establish a social media presence to help build relationships with younger generations, as well as increase awareness about health topics and promote healthy lifestyle practices (37). Collaborations between HCPs and influencers also presents an opportunity for connecting with younger generations and for promoting the distribution of reliable, evidence-based information (8).

### 4.5 Providing holistic and preventative health

Mental health and holistic care are priorities among younger generations (7), making it necessary to provide integrated mental and physical healthcare services. Despite mental health conditions being common among people with chronic physical health problems and being linked to age-related changes, older adults have inadequate access to mental health support services. Integrated approaches toward physical and mental health can ensure more comprehensive care for older generations and help promote healthy aging. Integrated care can also help to reduce mental health-associated stigma among older generations, by normalizing and increasing help-seeking to mental health support (38).

Although Gen Z are more likely than older generations to embrace preventative health, research indicates that Gen Z patients receive fewer HCP reminders regarding screenings and check-ups, and this cohort often lacks awareness about routinely offered preventive care services (39). Strategies are therefore needed to increase awareness of the availability of preventative care measures among this cohort.

## 5 Summary

There is growing pressure on healthcare systems due to an increasing prevalence of chronic disease and financial constraints. Healthcare burden is currently highest among older generations, but younger generations that are disengaged with traditional healthcare models will have increasing medical needs as they age. Innovative healthcare solutions can help to tackle the increasing healthcare burden and expectations for personalized, data-driven holistic care. Digital enablement will have a key role in the future of healthcare, with technologies that help to meet diverse generational needs, including hybrid care models, and AI-powered tools for diagnosis management, and preventative health. Digital health tools must be designed with end-users in mind. Successful integration is dependent on tackling challenges relating to digital health literacy, including tailored education initiatives to bridge the gap for less tech-savvy older generations. Ensuring equitable cross-generational access to relevant, high-quality medical content is also fundamental as misinformation represents a significant obstacle to patient empowerment. Generationally-tailored education and AI can deliver reliable information tailored to patients' literacy levels (19). Communication strategies should consider generational preferences and age-appropriate health messaging.

## Data Availability

The original contributions presented in the study are included in the article/supplementary material, further inquiries can be directed to the corresponding author.
